# Association of perinatal factors of epilepsy in very low birth weight infants, using a nationwide database in Japan

**DOI:** 10.1038/s41372-019-0494-7

**Published:** 2019-09-16

**Authors:** Yuki Matsushita, Yasunari Sakai, Michiko Torio, Hirosuke Inoue, Masayuki Ochiai, Kazuaki Yasuoka, Hiroaki Kurata, Junko Fujiyoshi, Masako Ichiyama, Tomoaki Taguchi, Kiyoko Kato, Shouichi Ohga, on behalf of the Neonatal Research Network of Japan (NRNJ)

**Affiliations:** 1 Department of Pediatrics, Graduate School of Medical Sciences, Kyushu University, Fukuoka, Japan; 2 Comprehensive Maternity and Perinatal Care Center, Kyushu University, Fukuoka, Japan; 3 Department of Perinatal and Pediatric Medicine, Graduate School of Medical Sciences, Kyushu University, Fukuoka, Japan; 4 Present Address: Department of Pediatrics, Clinical Research Institute, National Hospital Organization, Kyushu Medical Center, Fukuoka, Japan

**Keywords:** Epidemiology, Developmental biology, Neurophysiology

## Abstract

**Objective:**

To determine clinical features of very low birth weight infants (VLBWIs) who had developed epilepsy by age 3 years.

**Study design:**

Multicenter cohort study using the Neonatal Research Network of Japan database. We analyzed clinical variables of 8431 VLBWIs who had recorded data of neurological sequelae at age 3 years. Logistic regression identified the association between variables and development of epilepsy.

**Result:**

One hundred and forty-three (1.7%) infants developed epilepsy, 683 (8.1%) showed cerebral palsy (CP), and 1114 (13.2%) had psychomotor delay. Epilepsy was associated with history of sepsis [adjusted odds ratio (AOR) 3.23], severe intraventricular hemorrhage (IVH; AOR 5.13), and cystic periventricular leukomalacia (PVL; AOR 12.7). Severe IVH and cystic PVL were also frequently associated with CP and psychomotor delay.

**Conclusion:**

Severe IVH and cystic PVL are strongly associated with development of epilepsy, as well as other neurological sequelae, and are potential critical therapeutic targets.

## Introduction

Advances in perinatal–neonatal medicine have improved the survival rate of very low birth weight infants (VLBWIs), preterm-delivered infants weighing <1.5 kg at birth [1–4]. However, multicenter cohorts in Japan have shown that 8–15% of VLBWIs have cerebral palsy (CP) or delay in psychomotor development [5]. The developing brains of VLBWIs are thus considered vulnerable to environmental stress and carry a higher risk than brains of term infants for neurological sequelae.

Increased attention has been paid to epilepsy among the various sequelae of VLBWIs because preterm infants have greater susceptibility to the development of epilepsy in their later life [6]. Large population studies have been conducted in various countries [7, 8], although the prevalence of epilepsy among VLBWIs has not been precisely determined in Japan. The Neonatal Research Network of Japan (NRNJ) has conducted a nationwide cohort study of VLBWIs since 2003 [9, 10]. The NRNJ database contains all perinatal records from birth to 3–6 years of follow-up [11]. To date, however, perinatal factors associated with the development of neurological sequelae remain to be identified with high-powered studies. We therefore aimed to determine the prevalence rate and associated factors for epilepsy, as well as other neurological sequelae, in VLBWIs using the NRNJ database.

## Subjects and methods

### Study subjects

**Fig. 1:**
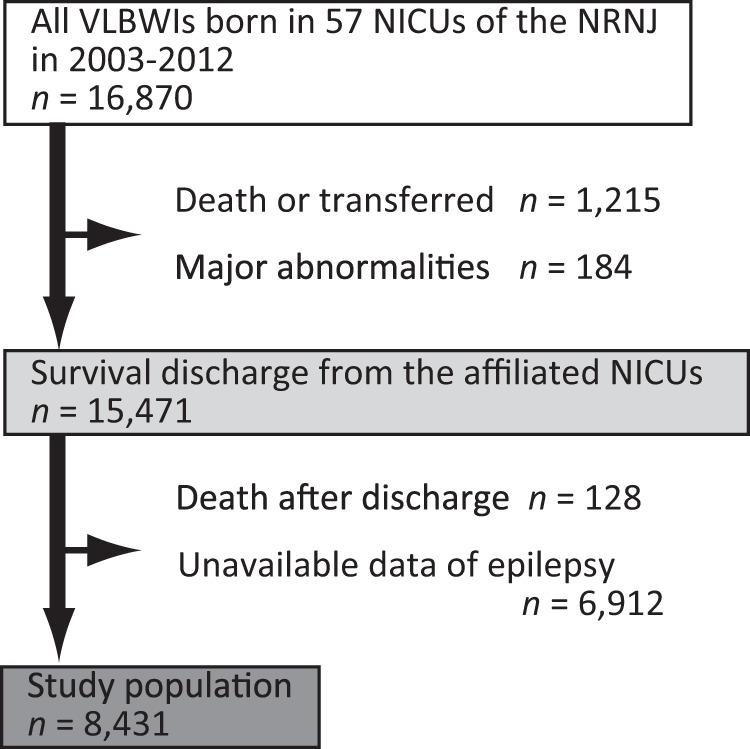
Very low birth weight infants (VLBWIs) enrolled in the present study. Among 16,870 VLBWIs who were born during 2003–2012, 15,471 (91.7%) were registered as survivors of the neonatal period. After discharge from hospital, 128 (0.8%) died by age 3 years. Medication records or other data for epilepsy were inaccessible for 6912 (40.9%) VLBWIs and these were excluded. A total of 8431 VLBWIs were registered as eligible for the present study

A total of 16,870 VLBWIs were born in 57 neonatal intensive care units (NICUs) that participated in a follow-up study of the NRNJ from 2003 to 2012 (Fig. 1). We excluded 184 (1.1%) infants who showed major congenital abnormalities: definitive chromosomal aberrancy and central nervous system disease of anencephaly, meningocele, fetal hydrocephaly and holoprosencephaly. A total of 1215 (7.2%) VLBWIs died or moved to external institutes before their discharge from these NICUs. Among the 15,471 survivors of the perinatal period, 6912 (41.0%) VLBWIs were excluded because they did not have recorded data for history of epilepsy. One hundred and twenty-eight (0.8%) children died between the time of discharge and 3 years of follow-up. Thus, 8431 VLBWIs had recorded data for epilepsy and other long-term neurological sequelae at age 3 years.

### Clinical data and variables

Clinical data included maternal complications and medication received during pregnancy and delivery. Independent variables were causative factors shown to affect neurological sequelae. Chorioamnionitis was diagnosed according to clinical findings when histological specimens were unavailable [12]. Antenatal corticosteroids were administered when preterm delivery was inevitable. Gestational age was calculated from the monthly ultrasound examination and the date of the last menstrual period. Small-for-gestational age was defined as birth weight below the 10th percentile of the Japanese birth size standard [13]. Moderate-to-severe bronchopulmonary dysplasia was defined as a respiratory disturbance receiving supplemental oxygen or positive pressure at gestational age of 36 weeks [14]. Patent ductus arteriosus was diagnosed by echocardiography and was treated by indomethacin or surgical ligation, when it was symptomatic and thought to be a possible contributor of medical state. Sepsis was defined as culture-proven septicemia or bacteremia during the hospital stay. Severe intraventricular hemorrhage (IVH) was defined as grade 3 or 4 according to the scale of Papile et al. [15]. Cystic periventricular leukomalacia (PVL) was defined as periventricular cyst formation. Necrotizing enterocolitis was diagnosed according to signs of pneumoperitoneum on radiographic examination [16]. Retinopathy of prematurity was diagnosed according to the international classification [17], and was treated by the laser/cryo-coagulation or intravitreal injection of an angiogenesis inhibitor.

### Neurological evaluation at age 3 years

Neurological evaluations were performed at age 3 years in accordance with a protocol of the Japanese Society for Follow-up Study of High-Risk Infants [5]. Trained testers performed developmental testing using the Kyoto Scale of Psychological Development 2001 or Enjoji Scale of Infant Analytical Development [18]. Developmental quotient (DQ) was obtained from the relative value of the developmental age to the chronological age, with the scale adjusted to 0–100. The criterion for psychomotor delay was set to DQ <70 [5]. CP was defined as a nonprogressive central nervous system disorder characterized by abnormal muscle tone in at least one extremity and uncontrollable movements or posture [19]. Epilepsy was defined as a history of recurrent seizures or the use of antiepileptic drugs [20]. VLBWIs with a history of neonatal seizure or febrile convulsion were not counted as those with epilepsy.

### Statistical analyses

All statistical analyses were performed using JMP^R^ 11 software (SAS Institute Inc., Cary, NC, USA). Continuous and discrete variables were analyzed using the Wilcoxon rank-sum test and the chi-squared test, respectively. A multiple logistic regression model yielded odds ratios (ORs) and 95% confidence intervals (CIs). Results with *P* < 0.05 were considered statistically significant.

## Results

### Clinical profiles of the study population

To test whether the enrolled subjects appropriately represented the parent population of VLBWIs, we first analyzed the demographic features of VLBWIs with (*n* = 8431) or without (*n* = 6912) available data for history of epilepsy (Table 1). Significant differences were observed in 13 of 20 items of the maternal complications or perinatal histories. The proportion of VLBWIs who developed CP (8.2% and 8.4%) or psychomotor delay (14.9% and 16.4%) did not differ in the rates between the two subpopulations, although those without data for epilepsy had missing data on CP and psychomotor delay. The clinical profiles of VLBWIs in our study were consistent with those in previous NRNJ studies [9, 10, 18, 21–24].

**Table 1:** Clinical characteristics of the eligible population

	Study population (*n* *=* 8431)			Not evaluated (*n* = 6912)				
Variables	^a^Total	Median/*n*	Range/%	^a^Total		Median/*n*	Range/%	*P*
^b^Gestational age [weeks, days] median, range	8424	28w6d	22w0d–39w1d	6905		29w0d	21w2d–40w1d	***<*** ***0.01***
^b^Birth weight [grams] median, range	8431	1066	267–1500	6911		1108	308–1500	***<*** ***0.01***
Maternal age of 35 or more years old	7937	2316	29.2	6522		1851	28.4	0.29
Multiple birth	8431	2128	25.2	6912		1748	25.3	0.94
Chorioamnionitis	8345	2250	27.0	6796		1656	24.4	***<*** ***0.01***
Antenatal steroid	8409	3857	45.9	6885		2704	39.3	***<*** ***0.01***
Cesarean section	8427	6733	79.9	6901		5236	75.9	***<*** ***0.01***
Male	8423	4337	51.5	6909		3542	51.3	0.78
Apgar score of less than 7-point at 5-minutes after birth	7914	1459	18.4	6353		1036	16.3	***<*** ***0.01***
Small-for-gestational age	8331	3333	40.0	6867		2621	38.2	***0.02***
Respiratory distress syndrome	8415	4736	56.3	6805		3387	49.8	***<*** ***0.01***
Moderate-to severe bronchopulmonary dysplasia	3133	1587	50.7	2258		1072	47.5	***0.02***
Sepsis	8411	590	7.0	6796		358	5.3	***<*** ***0.01***
Symptomatic patent ductus arteriosus	7093	3207	45.2	5252		2401	45.7	0.58
Severe intraventricular hemorrhage	8425	231	2.7	6895		240	3.5	***0.01***
Cystic periventricular leukomalacia	8424	255	3.0	6885		237	3.4	0.15
Necrotizing enterocolitis	8418	59	0.7	6798		70	1.0	***0.03***
Treating retinopathy of prematurity	8292	1247	15.0	6496		780	12.0	***<*** ***0.01***
Cerebral palsy	8317	683	8.2	2090		176	8.4	0.76
Developmental quotient of less than 70	7478	1114	14.9	1897		311	16.4	0.10

^a^Total number with available information

^b^Continuous variables are expressed as the median and range

Statistically significant *P*-values are in bold italics

### Incidence of neurodevelopmental sequealae

**Fig. 2:**
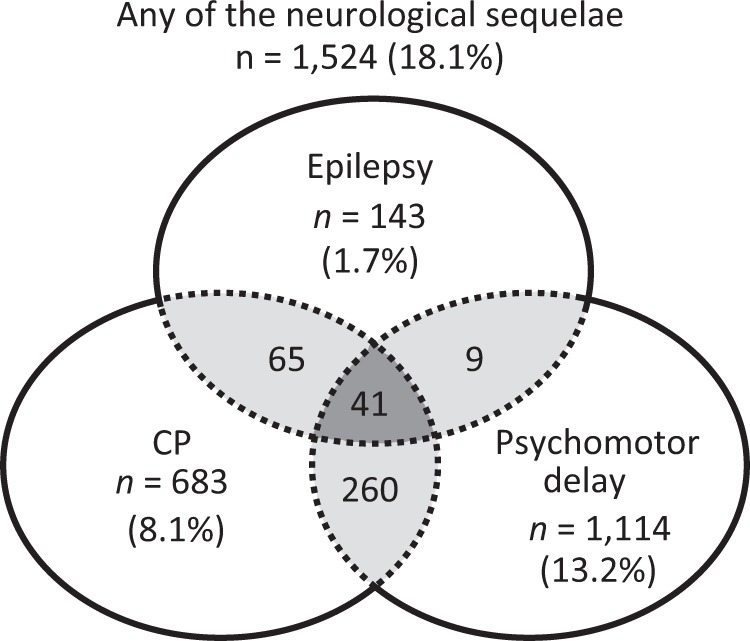
Prevalence of very low birth weight infants (VLBWIs) with cerebral palsy (CP), psychomotor delay, and epilepsy. The Venn diagram displays the number and prevalence rate (%) of VLBWIs with epilepsy, CP, and psychomotor delay among eligible subjects (*n* = 8431). In total, 1524 (18.1%) showed either of these neurological sequelae, while 41 (0.5%) developed all three sequelae by age 3 years

We determined the number of VLBWIs presenting with CP, psychomotor delay, or epilepsy among the study population (*n* = 8431, Fig. 1). Overall, 1524 (18.1%) VLBWIs had at least one of these neurological sequelae by age 3 years. Moreover, 1114 (13.2%) showed psychomotor delay, 683 (8.1%) had CP, and 143 (1.7%) developed epilepsy. Three hundred and seventy-five (4.4%) VLBWIs developed two or more of these sequelae in combination (Fig. 2).

### Perinatal risk factors associated with epilepsy and other neurological sequelae

To examine whether VLBWIs with epilepsy had a higher rate of perinatal complications than the whole population of VLBWIs, we performed univariate crude and multivariate adjusted analyses on the same subsets of variables as listed in Table 1. VLBWIs with epilepsy had perinatal history of sepsis (18.2%), severe IVH (28.7%), or cystic PVL (32.2%) at higher frequencies compared with the whole population of VLBWIs (*P* < 0.01, Table 2). Cystic PVL showed the highest adjusted OR (AOR) of 12.7 with 95% CI of 5.34–30.3. Sepsis (AOR 3.23; 95% CI 1.52–6.85) and severe IVH (AOR 5.13; 95% CI 2.10–12.5) were also ranked among the leading complications associated with onset of epilepsy in VLBWIs.

**Table 2:** Clinical factors associated with epilepsy (*n* = 143)

			Crude			Adjusted		
Variables	Median/*n*	Range/%	OR	95% CI	*P*	OR	95% CI	*P*
^a^Gestational age [weeks, days] median, range	29w3d	22w0d–37w1d	0.83	0.78–0.88	***<0.01***	0.89	0.60–1.32	0.55
^a^Birth weight [grams] median, range	870	321–1480	0.86	0.81–0.91	***<0.01***	0.98	0.95–1.02	0.30
Maternal age of 35 or more years old	27	21.3	0.65	0.42–1.00	0.05	0.48	0.20–1.16	0.10
Multiple birth	41	28.7	1.19	0.83–1.72	0.34	1.12	0.48–2.59	0.79
Chorioamnionitis	44	30.8	1.21	0.84–1.73	0.30	1.96	0.90–4.29	0.09
Antenatal steroid	60	42.0	0.85	0.61–1.19	0.34	0.67	0.33–1.35	0.26
Cesarean section	105	73.4	0.69	0.47–1.00	0.05	1.60	0.46–1.11	0.46
Male	89	62.2	1.56	1.11–2.20	***0.01***	1.91	0.90–4.02	0.09
Apgar score of less than 7-point at 5-minutes after birth	40	31.8	2.09	1.43–3.05	***<0.01***	0.83	0.59–1.18	0.92
Small-for-gestational age	46	32.2	0.71	0.50–1.01	0.05	1.45	0.40–5.23	0.57
Respiratory distress syndrome	99	69.2	1.76	1.23–2.52	***<0.01***	1.50	0.53–4.24	0.45
Moderate to severe bronchopulmonary dysplasia	49	66.2	1.94	1.19–3.15	***0.01***	1.85	0.84–4.05	0.12
Sepsis	26	18.2	3.04	1.97–4.68	***<0.01***	3.23	1.52–6.85	***<0.01***
Symptomatic patent ductus arteriosus	75	57.7	1.67	1.17–2.37	***<0.01***	0.65	0.29–1.42	0.28
Severe intraventricular hemorrhage	41	28.7	17.1	11.6–25.3	***<0.01***	5.13	2.10–12.5	***<0.01***
Cystic periventricular leukomalacia	46	32.2	18.3	12.6–26.7	***<0.01***	12.7	5.34–30.3	***<0.01***
Necrotizing enterocolitis	6	4.2	6.79	2.87–16.1	***<0.01***	3.02	0.60–15.1	0.18
Treating retinopathy of prematurity	48	33.8	2.96	2.08–4.21	***<0.01***	1.74	0.85–3.55	0.13

^a^Continuous variables are expressed as the median and range

Statistically significant *P*-values are in bold italics

### Common and unique risk factors for development of epilepsy in VLBWIs

We next investigated whether these three neonatal complications (cystic PVL, severe IVH, and sepsis) were uniquely identified risk factors for the development of epilepsy. We conducted identical tests for 1524 VLBWIs with psychomotor delay, CP, or epilepsy. Eight of the 18 perinatal variables were associated with the development of one of these three complications (Table S1). As expected from the results of Table 2, 192 (12.6%) of these VLBWIs showed cystic PVL, 158 (10.4%) had severe IVH, and 200 (13.2%) experienced sepsis during the neonatal period (Table S1). Statistical analysis revealed that these three complications were significantly associated with the development of any of the three neurological sequelae by age 3 years (*P* < 0.01).

Demographic analysis further identified 41 (0.48%) VLBWIs who developed all three sequelae in combination by age 3 years (Fig. 2). To gain further insight into their risk factors, we repeated the same analysis for those presenting with all three sequelae in combination, as formerly described (Table S2). A higher proportion of these VLBWIs experienced cystic PVL (39.0%) and severe IVH (26.8%) during the neonatal period than those with any of the neurological sequelae (12.6% and 10.4%, respectively; Table S1). These data indicated that cystic PVL and severe IVH were the most common and strongest associations for development of epilepsy, psychomotor delay, and CP in VLBWIs.

Lastly, we aimed to establish the unique risk factors for epilepsy. We analyzed further the demographic features of 1524 VLBWIs who showed either CP, psychomotor delay, or epilepsy. We subdivided the subjects into those with epilepsy (*n* = 143) and those without epilepsy (*n* = 1381). These two groups showed different comorbidity rates with CP and psychomotor delay. Compared with the Non-Epilepsy Group, the Epilepsy Group had higher comorbidity rates with CP (74.1% vs. 41.8%) but lower rates of psychomotor delay (35.0% vs. 77.0%). Both groups showed the highest AORs for development of neurological sequelae when they presented with cystic PVL (12.7 and 4.76) or severe IVH (5.13 and 2.95) (Fig. S1). None of these variables significantly differed in AORs between the two groups.

## Discussion

We clarified development of epilepsy by age 3 years among VLBWIs in Japan and investigated its risk factors. We identified, using the nationwide NRNJ database for VLBWIs, 143 (1.7%) infants who developed epilepsy. This percentage was higher than that among children in the general population (0.3–0.6%) [25, 26], and significantly higher than that in children with epilepsy onset age <3 years in Japan (0.6%) [27]. This indicates that various causes of brain injury contribute to the onset of epilepsy in preterm-delivered infants [28]. We also found that the prevalence rate was higher than that reported (1.0%) in an earlier nationwide cohort from Finland but lower than that reported in Turkey (5.6%) [7, 29]. In terms of birth weight, 43.7% (*n* = 3685) of infants had an extremely low birth weight <1 kg, and among them, 91 (2.5%) had epilepsy. This finding suggests that lower birth weight is associated with epilepsy onset by age 3 years in Japan. The results might have varied with differences in subpopulations of severely affected VLBWIs who survived the neonatal period, or with different ethical standards for the management of high-risk infants with multiple complications [26, 30].

Our study identified sepsis, severe IVH, and cystic PVL as the associations of epilepsy in VLBWIs. History of sepsis is also a known risk factor for the development of neonatal seizures and other comorbidities [31]. Among the clinical variables, the association between neonatal seizures and development of epilepsy has been shown in previous studies [29, 32]. However, we did not include neonatal seizures as one of the clinical variables because of ambiguous diagnoses with or without electroencephalography. Our data confirmed key findings in previous studies and that preterm infants with severe complications are at high risk for persistent neurological sequelae, including epilepsy. In our subjects, the comorbidity rate with psychomotor delay was lower in the Epilepsy Group than that in the Non-Epilepsy Group. Our extensive multivariate analyses further demonstrated that severe IVH and cystic PVL were recurrent risk factors for development of the other two sequelae, CP and psychomotor delay. This confirms that we were unable to detect unique predisposing factors that were solely associated with onset of epilepsy.

Among 1524 VLBWIs with at least one sequela, 375 (24.6%) showed more than two. We identified multiple perinatal variables that were overly represented among VLBWIs with psychomotor delay, CP, or epilepsy. Although we applied multivariate analysis to identify explanatory variables, it merely confirmed that severely affected infants were more likely to show unfavorable neurological outcomes than were those with milder distress at birth [33]. It remains to be clarified whether these risk factors place differential weight on neurological outcomes of VLBWIs. Therefore, a scoring system will provide clues for characterizing more accurately those with a risk of developing epilepsy, in addition to other neurodevelopmental sequelae [34].

The strength of our study was that we used a nationwide cohort delineating the neurodevelopmental profiles for a whole population of VLBWIs in Japan over a 10-year period. To provide comprehensive data from eligible subjects, we aimed to determine the optimal age to investigate their neurodevelopmental profiles. By setting it to 3 years, we clarified that 8.2% and 14.9% of VLBWIs (*n* = 8431) developed CP and psychomotor delay, respectively, and we regard these results as reasonable. We considered that 3 years was an appropriate age at which to evaluate neurological comorbidities because there were too-high drop-off rates at older ages. In contrast, we understand that neurodevelopmental deficits might further evolve in preschool and school-age children [35]. More accurate profiles in neurocognitive functions of VLBWIs will therefore be unveiled through pursuing their educational performance and achievements.

We recognize several limitations to the present study. First, the follow-up rate was only 54.8% among 15,471 potentially eligible subjects. In Japan, there is a traditional perinatal support system for a pregnant woman. She returns to her parents’ home before labor, delivers at a hospital near her hometown, and the baby grows up with parental support. Meanwhile, a follow-up study is conducted by a doctor at the NICU where each infant was under medical care. In cases where the woman has moved from her hometown after hospital discharge, researchers could be at a risk of missing the subject. Many pregnant women choose this practice of delivery in their hometown, and their infants are raised in the NICU away from the place where they will grow up afterward. Thus, the NRNJ studies have had a high percentage (43–63%) of drop-outs [22–24]. We did not choose earlier ages for evaluation because too short a duration of follow-up may have compromised the accuracy of estimating the prevalence of children with epilepsy. We therefore considered that our data fully disclosed the neurological features of VLBWIs with the currently available dataset from the NRNJ database. In addition, there was a lack of data for CP and developmental delay in the excluded population. In those without epilepsy data (*n* = 6912), only 2090 had data for CP, and only 1897 had data for psychomotor delay. Consequently, the study population may not be representative of the whole population. The percentages of sepsis and IVH were significantly different between the patients with and without epilepsy data. Those two variables implied a possibility to deviate the analysis in our study; however, we were not able to evaluate whether the study population had more severe disability than the excluded group. The other limitation was the lack of detailed information about epilepsy: age of onset, diagnostic categories, medication, and clinical course of epileptic seizures. Their correlations with findings in electroencephalography or neuroimaging were not documented either. These data will further characterize the nature of pathogenic insults to the brain of VLBWIs.

In conclusion, the NRNJ database revealed that severe IVH, cystic PVL, and sepsis were critical associations for the onset of epilepsy in VLBWIs by age 3 years. Severe IVH and cystic PVL were also associated with the development of other sequelae, CP and psychomotor delay. These data will serve as a reference for neonatologists to identify therapeutic targets and improve outcomes of neurological morbidity in high-risk VLBWIs [21]. With regard to epilepsy specific for VLBWIs, details of its characterization, onset age, classification, and severity should be described in a future study.

## Supplementary information

Supplementary FigureS1

Supplementary TableS1

Supplementary TableS1
