# Supplementary material for: Association of perinatal factors of epilepsy in very low birth weight infants, using a nationwide database in Japan
Source: J Perinatol. 2019 Sep 16;39(11):1472–9. doi: 10.1038/s41372-019-0494-7 (PMC6892414; doi:10.1038/s41372-019-0494-7)
Supplement: Supplementary file 1 — Supplementary FigureS1 [file 41372_2019_494_MOESM1_ESM.pdf]

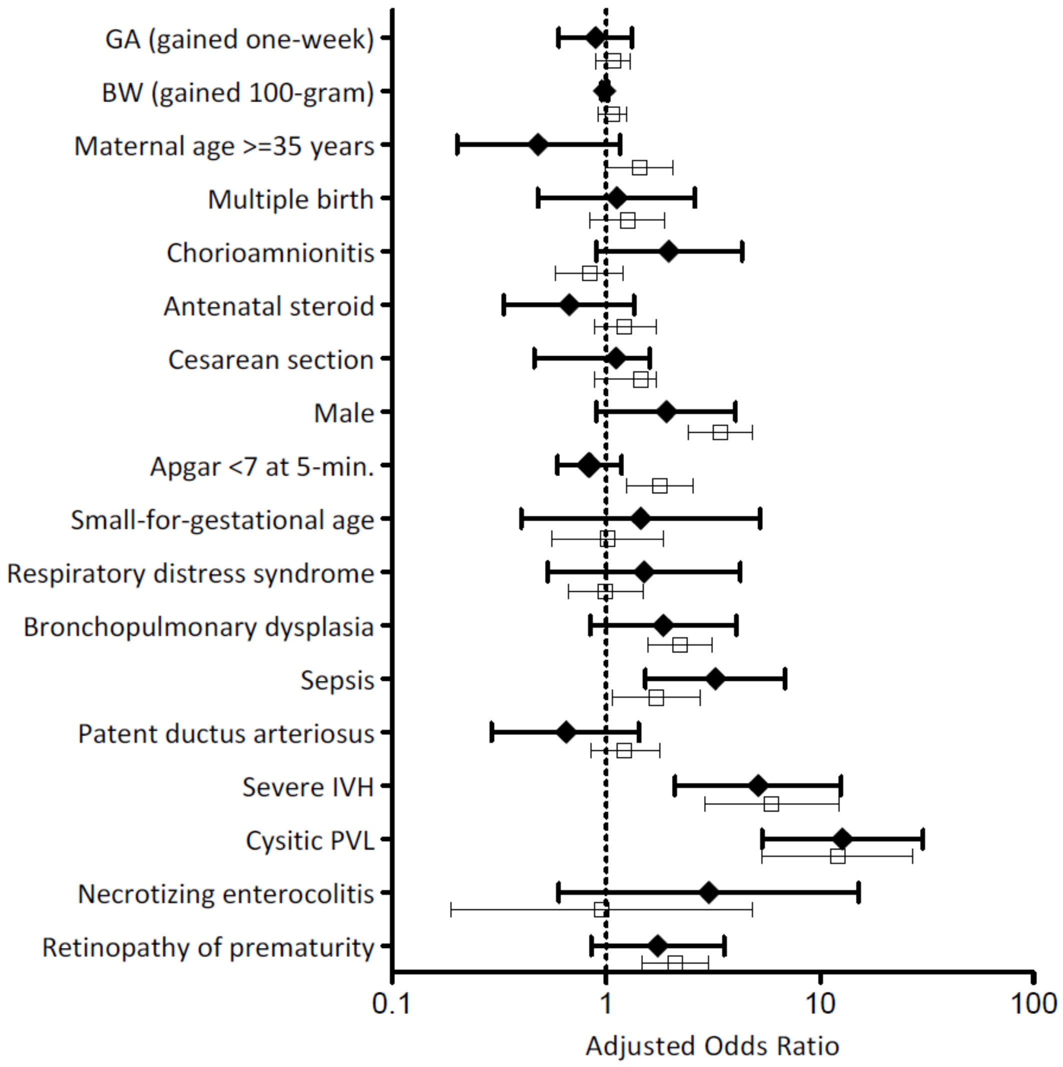

Figure S1 Comparative analysis on the associations for the development of epilepsy and other neurological sequelae.

The forest plot presents the adjusted OR and 95% CI (horizontal lines) of the indexed clinical profiles for VLBWIs with epilepsy (n = 143, Epilepsy group) and those without epilepsy (n = 1,381, non-Epilepsy group). Filled diamonds indicate the OR of the profiles for Epilepsy group, and outlined squares for non-Epilepsy group. Relative values to those of the whole VLBWIs (n = 8,431) are shown as logarithmic scales. Note that cystic periventricular leukomalacia (PVL) and grade 3 or 4 intraventricular hemmorhage (severe IVH) are the common complications that provided the highest values in OR for both Epilepsy and non-Epilepsy groups. y/o, years old; GA, gestational age; and BW, birth weight.
