# Supplementary material for: Association of perinatal factors of epilepsy in very low birth weight infants, using a nationwide database in Japan
Source: J Perinatol. 2019 Sep 16;39(11):1472–9. doi: 10.1038/s41372-019-0494-7 (PMC6892414; doi:10.1038/s41372-019-0494-7)
Supplement: Supplementary file 2 — Supplementary TableS1 [file 41372_2019_494_MOESM2_ESM.docx]

|  |  |  | **Crude** | | | | |  | **Adjusted** | | | | |
| --- | --- | --- | --- | --- | --- | --- | --- | --- | --- | --- | --- | --- | --- |
| **Variables** | **Median / *n*** | **Range / %** | **OR** | **95% CI** | | | ***P*** |  | **OR** | **95% CI** | | | ***P*** |
| *Gestational age [weeks, days] median, range | 27w0d | 22w0d - 38w1d | 0.80 | 0.79 | - | 0.82 | ***<0.01*** |  | 0.97 | 0.85 | - | 1.10 | 0.60 |
| *Birth weight [grams] median, range | 870 | 276 - 1,500 | 0.82 | 0.80 | - | 0.84 | ***<0.01*** |  | 0.89 | 0.80 | - | 0.99 | ***0.04*** |
| Maternal age of 35 or more years old | 417 | 28.9 | 0.98 | 0.87 | - | 1.12 | 0.78 |  | 1.12 | 0.87 | - | 1.44 | 0.38 |
| Multiple birth | 357 | 23.4 | 0.90 | 0.79 | - | 1.03 | 0.12 |  | 1.04 | 0.78 | - | 1.38 | 0.79 |
| Chorioamnionitis | 503 | 33.5 | 1.45 | 1.28 | - | 1.63 | ***<0.01*** |  | 0.80 | 0.63 | - | 1.03 | 0.09 |
| Antenatal steroid | 724 | 47.7 | 1.06 | 0.95 | - | 1.18 | 0.33 |  | 0.98 | 0.78 | - | 1.23 | 0.84 |
| Cesarean section | 1201 | 78.9 | 0.92 | 0.80 | - | 1.06 | 0.23 |  | 1.20 | 0.84 | - | 1.71 | 0.32 |
| Male | 979 | 64.3 | 1.94 | 1.73 | - | 2.18 | ***<0.01*** |  | 2.32 | 1.82 | - | 2.94 | ***<0.01*** |
| Apgar score of less than 7-point at 5-minutes after birth | 443 | 32.2 | 2.56 | 2.25 | - | 2.93 | ***<0.01*** |  | 1.34 | 1.04 | - | 1.73 | ***0.02*** |
| Small-for-gestational age | 478 | 32.0 | 0.66 | 0.59 | - | 0.75 | ***<0.01*** |  | 0.87 | 0.57 | - | 1.32 | 0.50 |
| Respiratory distress syndrome | 1074 | 70.6 | 2.10 | 1.86 | - | 2.37 | ***<0.01*** |  | 0.85 | 0.64 | - | 1.13 | 0.26 |
| Moderate to severe bronchopulmonary dysplasia | 516 | 61.7 | 1.90 | 1.62 | - | 2.25 | ***<0.01*** |  | 1.67 | 1.32 | - | 2.12 | ***<0.01*** |
| Sepsis | 200 | 13.2 | 2.66 | 2.21 | - | 3.21 | ***<0.01*** |  | 1.70 | 1.22 | - | 2.36 | ***<0.01*** |
| Symptomatic patent ductus arteriosus | 783 | 60.0 | 2.02 | 1.79 | - | 2.29 | ***<0.01*** |  | 0.92 | 0.71 | - | 1.19 | 0.53 |
| Severe intraventricular hemorrhage | 158 | 10.4 | 11.2 | 8.32 | - | 15.1 | ***<0.01*** |  | 6.15 | 3.46 | - | 10.9 | ***<0.01*** |
| Cystic periventricular leukomalacia | 192 | 12.6 | 15.5 | 11.5 | - | 21.0 | ***<0.01*** |  | 13.0 | 6.53 | - | 26.0 | ***<0.01*** |
| Necrotizing enterocolitis | 32 | 2.1 | 5.90 | 3.42 | - | 10.2 | ***<0.01*** |  | 1.56 | 0.50 | - | 4.81 | 0.44 |
| Treating retinopathy of prematurity | 423 | 28.1 | 2.87 | 2.50 | - | 3.29 | ***<0.01*** |  | 1.65 | 1.29 | - | 2.12 | ***<0.01*** |

**Table S1** Clinical factors associated with any of the neurological sequelae (*n* = 1,524)

*Continuous variables are expressed as the median and range.
