# Supplementary material for: Association of perinatal factors of epilepsy in very low birth weight infants, using a nationwide database in Japan
Source: J Perinatol. 2019 Sep 16;39(11):1472–9. doi: 10.1038/s41372-019-0494-7 (PMC6892414; doi:10.1038/s41372-019-0494-7)
Supplement: Supplementary file 3 — Supplementary TableS1 [file 41372_2019_494_MOESM3_ESM.docx]

**Table S2** Clinical factors associated with all the three sequelae in combination (*n* = 41)

|  |  |  | **Crude** | | | | |  | **Adjusted** | | | | |
| --- | --- | --- | --- | --- | --- | --- | --- | --- | --- | --- | --- | --- | --- |
| **Variables** | **Median / *n*** | **Range / %** | **OR** | **95% CI** | | | ***P*** |  | **OR** | **95% CI** | | | ***P*** |
| *Gestational age [weeks, days] median, range | 27w4d | 22w3d - 35w3d | 0.87 | 0.78 | - | 0.97 | ***0.01*** |  | 0.89 | 0.40 | - | 1.96 | 0.77 |
| *Birth weight [grams] median, range | 904 | 363 - 1,480 | 0.89 | 0.80 | - | 0.98 | ***0.02*** |  | 0.88 | 0.45 | - | 1.73 | 0.71 |
| Maternal age of 35 or more years old | 9 | 24.3 | 0.78 | 0.37 | - | 1.65 | 0.51 |  | 0.52 | 0.10 | - | 2.88 | 0.46 |
| Multiple birth | 12 | 29.3 | 1.23 | 0.62 | - | 2.41 | 0.55 |  | 1.20 | 0.27 | - | 5.36 | 0.81 |
| Chorioamnionitis | 8 | 19.5 | 0.66 | 0.30 | - | 1.42 | 0.28 |  | 0.59 | 0.12 | - | 2.80 | 0.51 |
| Antenatal steroid | 21 | 51.2 | 1.24 | 0.67 | - | 2.29 | 0.49 |  | 1.23 | 0.31 | - | 4.79 | 0.77 |
| Cesarean section | 28 | 68.3 | 0.54 | 0.28 | - | 1.04 | 0.06 |  | 0.99 | 0.09 | - | 11.2 | 0.99 |
| Male | 28 | 68.3 | 2.04 | 1.05 | - | 3.94 | ***0.03*** |  | 1.81 | 0.42 | - | 7.72 | 0.42 |
| Apgar score of less than 7-point at 5-minutes after birth | 10 | 27.8 | 1.71 | 0.82 | - | 3.55 | 0.15 |  | 1.52 | 0.32 | - | 7.29 | 0.60 |
| Small-for-gestational age | 15 | 36.6 | 0.86 | 0.46 | - | 1.63 | 0.65 |  | 1.21 | 0.10 | - | 14.2 | 0.88 |
| Respiratory distress syndrome | 30 | 73.2 | 2.13 | 1.06 | - | 4.25 | ***0.03*** |  | 1.38 | 0.15 | - | 13.0 | 0.78 |
| Moderated to severe bronchopulmonary dysplasia | 9 | 50.0 | 0.97 | 0.39 | - | 2.46 | 0.96 |  | 1.32 | 0.32 | - | 5.46 | 0.70 |
| Sepsis | 3 | 7.3 | 1.05 | 0.32 | - | 3.40 | 0.94 |  | 0.64 | 0.09 | - | 4.47 | 0.65 |
| Symptomatic patent ductus arteriosus | 25 | 69.4 | 2.77 | 1.36 | - | 5.63 | ***<0.01*** |  | 1.49 | 0.27 | - | 8.30 | 0.65 |
| Severe intraventricular hemorrhage | 11 | 26.8 | 13.6 | 6.73 | - | 27.5 | ***<0.01*** |  | 11.6 | 2.32 | - | 57.5 | ***<0.01*** |
| Cystic periventricular leukomalacia | 16 | 39.0 | 21.8 | 11.5 | - | 41.4 | ***<0.01*** |  | 10.2 | 2.22 | - | 47.2 | ***<0.01*** |
| Necrotizing enterocolitis | 3 | 7.3 | 11.7 | 3.52 | - | 39.1 | ***<0.01*** |  | 10.7 | 0.70 | - | 162 | 0.09 |
| Treating retinopathy of prematurity | 11 | 27.5 | 2.15 | 1.07 | - | 4.32 | ***0.03*** |  | 0.42 | 0.08 | - | 2.12 | 0.30 |

*Continuous variables are expressed as the median and range.
